# Clarifying the nomenclatural history of *Tovomitopsis*, a Brazilian endemic genus of Clusiaceae

**DOI:** 10.3897/phytokeys.181.70745

**Published:** 2021-09-01

**Authors:** Lucas C. Marinho, Pedro Fiaschi, André M. Amorim, Volker Bittrich

**Affiliations:** 1 Universidade Federal do Maranhão, Departamento de Biologia, São Luís, MA, Brazil Universidade Federal do Maranhão São Luís Brazil; 2 Universidade Estadual de Feira de Santana, Programa de Pós-Graduação em Botânica, Feira de Santana, BA, Brazil Universidade Estadual de Feira de Santana Feira de Santana Brazil; 3 Universidade Federal de Santa Catarina, Departamento de Botânica, Florianópolis, SC, Brazil Universidade Federal de Santa Catarina Florianópolis Brazil; 4 Universidade Estadual de Santa Cruz, Departamento de Ciências Biológicas, Ilhéus, BA, Brazil Universidade Estadual de Santa Cruz Ilhéus Brazil; 5 Rua Mario de Nucci, Campinas, SP, Brazil Unaffiliated Campinas Brazil

**Keywords:** Atlantic Forest, lectotype, Malpighiales, Neotropics, South America

## Abstract

*Tovomitopsis* Planch. & Triana is a Brazilian Atlantic Forest endemic genus composed of two species: *T.paniculata* (Spreng.) Planch. & Triana and *T.saldanhae* Engl. An investigation was conducted to clarify the nomenclatural history of *Tovomitopsis*. We report the results of this investigation, provide an updated description of the genus, and propose lectotypes for *T.paniculata* and its synonyms: *Tovomitafoliosa* C.Presl and *Tovomitapaniculata* Cambess. We also propose lectotypes for *T.saldanhae* and for the new synonym *Clusiaangustifolia* Engl.

## Introduction

*Tovomitopsis* Planch. & Triana is a Brazilian endemic genus currently composed of two species: *T.paniculata* (Spreng.) Planch. & Triana and *T.saldanhae* Engl. Both species occur in preserved remnants of Atlantic Forest in southeastern Brazil (Marinho 2021). *Tovomitopsis* was proposed in 1860 (Planchon and Triana 1860) as a replacement name for the illegitimate *Bertolonia* Spreng. (non *Bertolonia*Raddi 1820) and to accommodate presumably tetramerous flowered species. Together with *Chrysochlamys* Poepp. and *Tovomita* Aubl., these three genera were known by Planchon and Triana (1860: 225) as *Les Tovomitées*, being differentiated from each other especially by the arrangement of sepals on the floral bud: in *Chrysochlamys* and *Tovomitopsis* the outer sepals are smaller, exposing the inner sepals in bud, while in *Tovomita* the outer sepals are larger, covering the inner sepals and petals. Moreover, Planchon and Triana (1860) indicated that aril anatomy could be useful to differentiate, or at least, better circumscribe *Les Tovomitées*, but surprisingly this topic has not yet been further investigated.

Although some recent studies still indicate floral merosity as relevant to distinguish *Tovomitopsis* from *Chrysochlamys* (e.g. Hammel 2010), the latter includes species with four or five petals (e.g. Hammel 1999; Martínez y Pérez et al. 2015), and a clear morphological distinction between these two genera is yet missing. Taxonomic treatments and checklists carried out in Mexico (Martínez y Pérez et al. 2015), Central America (e.g. Hammel 1999), northern South America (Kearns 1998), where *Chrysochlamys* is distributed, and the Brazilian Atlantic Forest (Oliveira-Filho 2006) considered these two genera as congeneric.

Molecular phylogenetic evidence shows *Tovomitopsis* in a politomy with *Dystovomita* and the rest of Clusieae, and thus not very closely related to *Chrysochlamys* despite their gross morphological similarity (Marinho et al. 2019). Pollen morphology and aril anatomy (Planchon and Triana 1860; Hammel 1999; Stevens 2007; Marinho et al. 2019) have been suggested as promising to distinguish these two genera, but were so far gathered from only a few species of *Chrysochlamys*. The presence of resin glands in the anther dorsal region of *Tovomitopsis* could be a synapomorphy of the genus, and the absence of a pistillode in staminate flowers of *Chrysochlamys* could be also relevant to distinguish these genera (Bittrich and Marinho pers. com.).

*Tovomitopsis* consists of dioecious small trees or shrubs with prop roots and yellowish viscous exudate. The opposite leaves are petiolate, entire, chartaceous or coriaceous, with numerous closely arranged veins. The flowers have two pairs of sepals, the outer ones being smaller than the inner ones, and two pairs of whitish petals. Staminate flowers have yellow subclavate resiniferous stamens and a pistillode; pistillate flowers have staminodes similar to the stamens and a green-yellowish pistil with expanded stigmas. The fruits are green fleshy capsules that expose seeds with an orange vascularized aril when ripe (Bittrich 2003; Stevens 2007).

Although *Tovomitopsis* includes only two species, the genus has a long taxonomic history (see Hammel 1999), with several species floating among the three genera of *Les Tovomitées* (sensu Planchon and Triana 1860). The type species of the genus, *Tovomitopsispaniculata*, was described two hundred years ago, but a few nomenclatural issues remain to be addressed. Here, we clarify the nomenclatural history of *Tovomitopsis*, provide an updated description for the genus, and propose lectotypes for *T.paniculata* and its synonyms: *Tovomitafoliosa* C.Presl and *Tovomitapaniculata* Cambess. We also propose lectotypes for *T.saldanhae* and its new synonym, *Clusiaangustifolia* Engl.

## Material and methods

This study is based on the analysis of the protologues of *Tovomitopsis* names and some of its synonyms, on visits to historical collections in Europe (B, K, M, P, W) and the Americas (A, GH, NY, R, RB; herbaria acronyms according to Thiers 2021), and by analyzing specimens from virtual herbaria. Data on collectors and botanists were accessed in the Taxonomic Literature II website (Stafleu and Cowan 1976–1988). All nomenclatural decisions follow the International Code of Nomenclature for algae, fungi, and plants (Turland et al. 2018).

## Nomenclature and discussion

**Figure 1.**
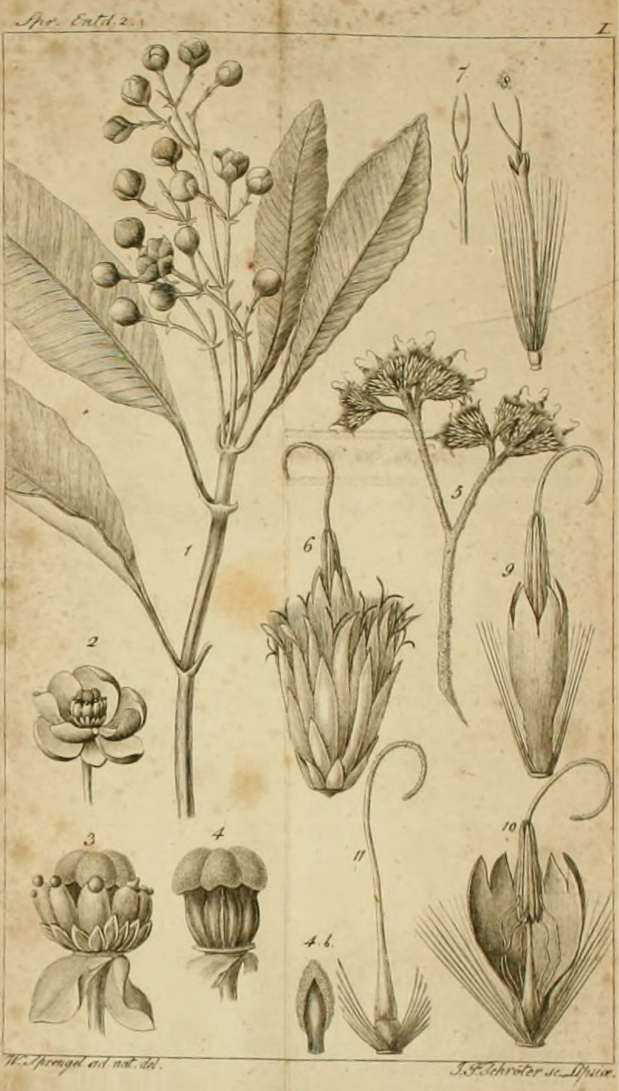
Lectotype (1–4b) of *Bertoloniapaniculata* Spreng. (≡ *Tovomitopsispaniculata* (Spreng.) Planch. & Triana) published by Sprengel (1821) in “Neue Entdeckungen im ganzen Umfang der Pflanzenkunde II”.

*Tovomitopsis* was proposed by Planchon and Triana (1860) as a replacement name for *Bertolonia* Spreng. [with just one species, *B.paniculata*, which was initially attributed to Chenopodiaceae (as “Chenopodieae”)], a later homonym of *Bertolonia* Raddi (Melastomataceae). Along with the newly transferred *T.paniculata* they described five additional new species, all six of which they felt could be distinguished from *Chrysochlamys* and *Tovomita*. *Bertoloniapaniculata* Spreng. was based on a pistillate specimen according to the illustration provided by the author (see Fig. 1, 1–4b). Sprengel (1821) did not mention either location, number or collector name for this collection.

**Figure 2.**
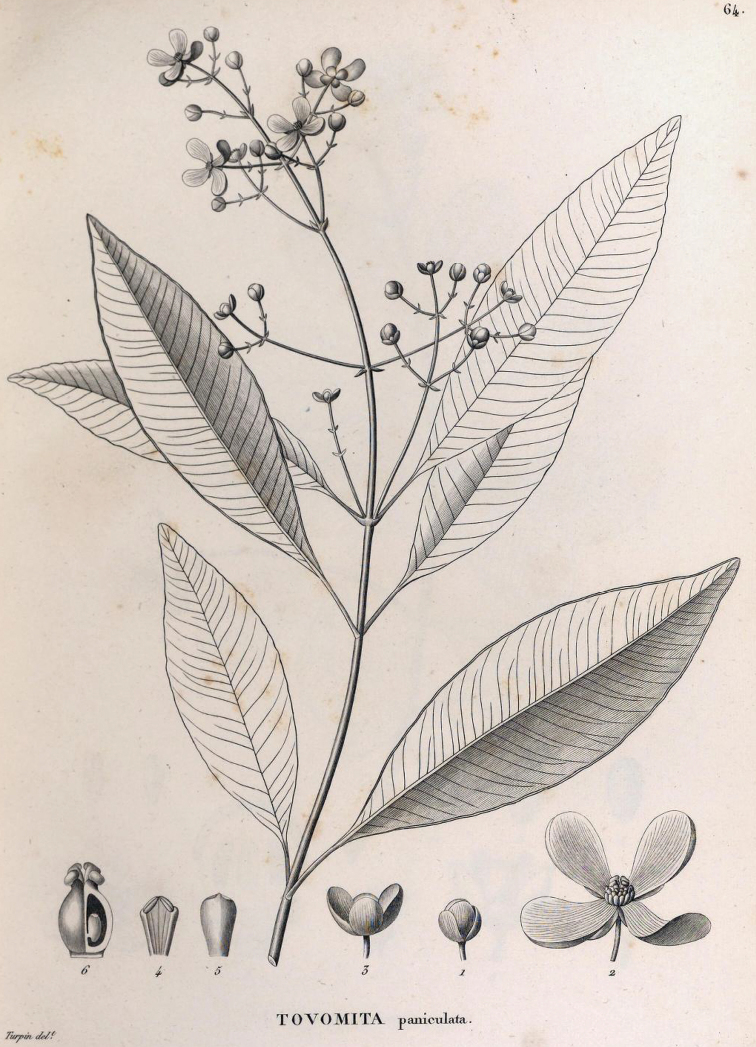
Illustration of *Tovomitapaniculata* Cambess. published by Cambessèdes (1828) in “Flora Brasiliae Meridionalis”.

In the “Flora Brasiliae Meridionalis”, edited by Auguste de Saint-Hilaire et al., Jacques Cambessèdes (1828) used the same epithet “paniculata” when he published the new species *Tovomitapaniculata* Cambess. This binomial is sometimes mistakenly interpreted as a new combination for *Bertoloniapaniculata* Spreng. However, Cambessèdes is clearly indicated as the author of the Guttiferae monograph at the end of the treatment, and the † sign was used to indicate a new species throughout “Flora Brasiliae Meridionalis”. The complete description of *Tovomitapaniculata* that Cambessèdes (1828) provided included stamens, pollen grains and gynoecium, indicating that he studied both staminate and pistillate specimens. The protologue contains the following statement: “*in sylvis primaevis propè vicum Aguassu, haud longè ab urbe Rio de Janeiro. Florebat Februario*”, and an illustration of a branch with many flowers, a feature found only in staminate specimens, while the illustration details show a pistillate flower and the detail of an ovary, as well as stamens with well-formed anthers (see Fig. 2). Cambessèdes (1828) did not indicate a type collection, but one specimen housed at P that was collected by Saint-Hilaire is probably the original material. The specimen P00093861 bears a label indicating the same location as in the protologue.

Presl (1834) proposed *Tovomitafoliosa* C.Presl, as a new species and provided a detailed description and an illustration, but again without indication of the material he used. The author cited only “*Habitat in Brasilia ad Rio de Janeiro*”. Although he described the species as a *Tovomita*, the pair of outer sepals not covering the inner sepals and other floral parts allow us to recognize a *Tovomitopsispaniculata* specimen in the illustration. In 1860, Planchon and Triana described *Tovomitopsis* and included, in addition to *Tovomitopsispaniculata* (Spreng.) Planch. & Triana (≡ *Bertoloniapaniculata* Spreng.), five new species (currently placed in *Chrysochlamys*), and justified this decision based, in part, on the number of floral parts: while *Tovomitopsis* was circumscribed to include tetramerous flowers, *Chrysochlamys* retained the species with pentamerous flowers. In that work, the authors indicated two specimens for *T.paniculata*: “Brésil, Rio-de-Janeiro (Aug. de Saint-Hilaire; Sellow)”.

In the “Flora brasiliensis”, Engler (1888) provided a description and an illustration of *Tovomitopsispaniculata* citing eight collections, all of which were collected in Rio de Janeiro state: 1 – *Habitat in Brasiliae prov. Rio de Janeiro: Sello in herb. reg. Berol.*; 2 and 3 – *Glaziou n. 7429, 12466*; 4 – *in silvis primaevis pr. Aguasta: St-Hilaire*; 5 – *in silvis umbrosis Capivary: Riedel n. 1318 in herb. Petrop.*; 6 – *in Serra de Friburgo: Saldanha n. 7044*; 7 – *in Serra de Tingua: Saldanha n. 5313*; 8 – *in Serra dos Orgãos: Riedel – Flor. Febr.* The author also described two new species in the genus: *Tovomitopsisspruceana* Engl., based on specimens from the Peruvian Amazon, and *Tovomitopsissaldanhae* Engl., based on specimens from Rio de Janeiro state.

The material used in the original description of *Tovomitopsispaniculata* was not mentioned or indicated by either Planchon and Triana or Engler, but the protologue and subsequent publications provided two important clues to find the type: i) both the description of the pistil and the illustration point to a pistillate specimen; ii) several points indicate that the type was collected in Rio de Janeiro state. For the locality of his *Tovomitapaniculata*, Cambessèdes (1828) mentioned the village of *Aguassu*, which we believe to be Nova Iguaçu (before spelled as *Iguassú*), today a municipality in the metropolitan region of Rio de Janeiro city. Planchon and Triana (1860) cited Rio de Janeiro as the only locality of *Tovomitopsispaniculata*, and also all specimens cited by Engler (1888) came from the state of Rio de Janeiro. Both Planchon and Triana (1860) and Engler (1888) cited specimens collected by Friedrich Sellow and Auguste de Saint-Hilaire as the basis for their respective descriptions. Sellow collected in the state of Rio de Janeiro between 1814 and 1821 (Stafleu and Cowan 1976–1988) and most of his specimens were deposited in B, but there are additional specimens originally from the Müller-Sprengel herbarium which were purchased by B in 1890 (Stafleu and Cowan 1976–1988). We speculate that Kurt Sprengel, the German botanist who first described *Tovomitopsispaniculata* (as *Bertoloniapaniculata*), likely studied one or more specimens collected by Sellow rather than the ones collected by Saint-Hilaire.

During a visit to European herbaria in 2016, a search at B was made for specimens assigned to the names *Bertoloniapaniculata*, *Tovomitapaniculata*, and *Tovomitopsispaniculata*, but none were found. Likewise, there are no Macbride negatives of such specimens in the Chicago Field Museum. Two relevant Sellow specimens were located in K (K001231050, image seen in Reflora Virtual Herbarium 2021), with the ♂ symbol indicating that it is a staminate specimen, and US (01882513) herbaria, both with floral buds; however, it is uncertain that these specimens are duplicates of a presumed type specimen housed in B. Therefore, we selected the illustration provided by Sprengel (1821) as the lectotype of *Tovomitopsispaniculata*, as this is the only unambiguous original material known to us. We also choose the illustration provided by Presl (1834) as the lectotype of *Tovomitafoliosa*; and followed Planchon and Triana (1860) and Engler (1888) by considering *T.paniculata* Cambess. as an heterotypic synonym of *Tovomitopsispaniculata*, rather than a new combination.

### Tovomitopsis paniculata

Taxon classificationPlantaeMalpighialesClusiaceae

(Spreng.) Planch. & Triana, Ann. Sci. Nat. Bot., Sér. 4, 14: 262. 1860.

7C94B441-AD27-57DE-9AE9-F76498EF411F

**Figure 3.**
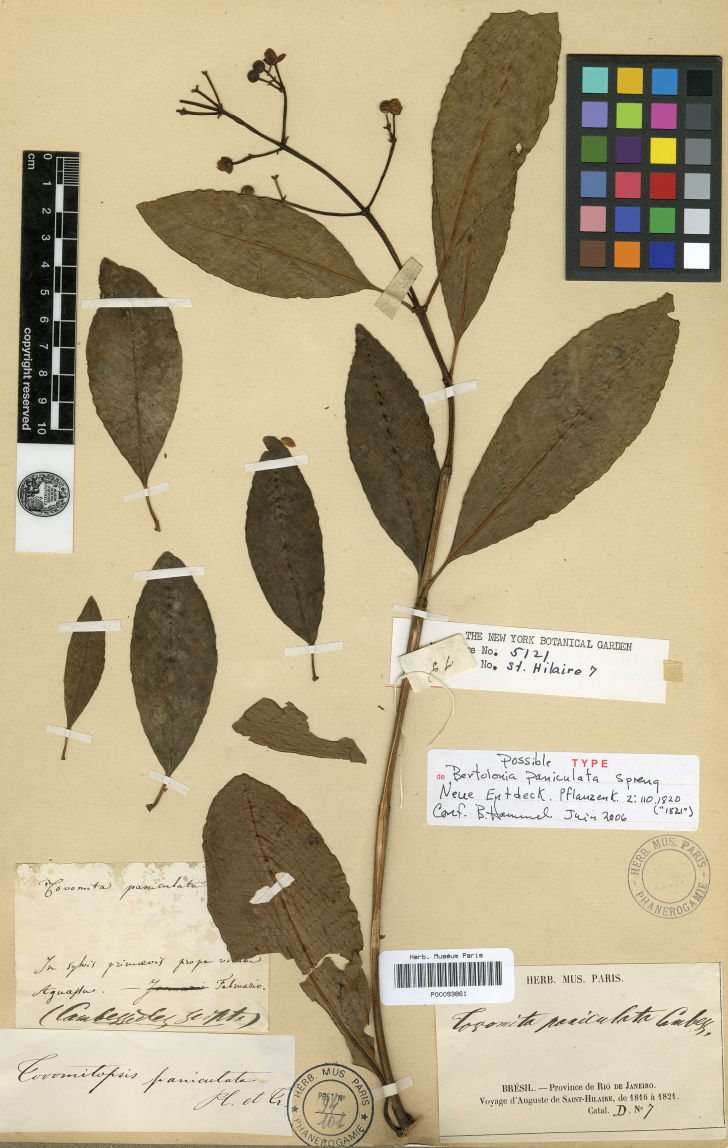
Lectotype of *Tovomitapaniculata* Cambess. (P00093861) housed at P.

**Figure 4.**
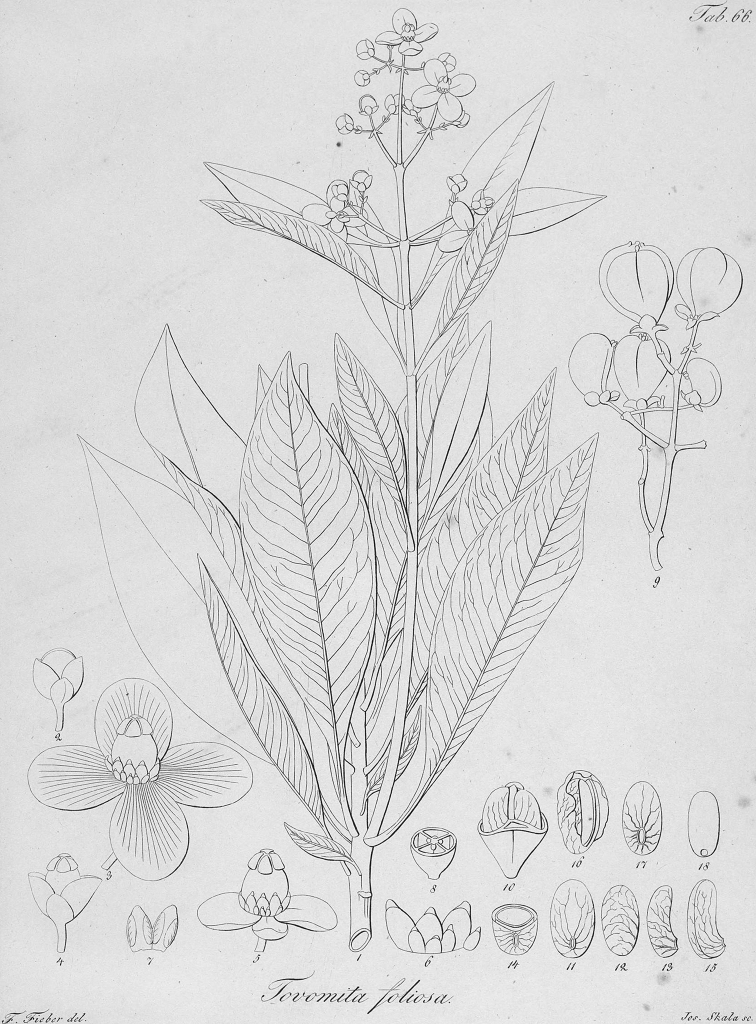
Lectotype of *Tovomitafoliosa* C.Presl (= *Tovomitopsispaniculata* (Spreng.) Planch. & Triana) published by Presl (1834) in "*Symbolae botanicae, sive, Descriptiones et icones plantarum novarum aut minus cognitarum*".

≡ Bertoloniapaniculata Spreng., Neue Entdeck. Pflanzenk. II: 110, t. I. 1820 (“1821”).
= Tovomitapaniculata Cambess., Fl. Bras. Merid. (quarto ed.) 1(8): 315, pl. 64. 1828. Type. lectotype (designated here), [Brazil: Rio de Janeiro] in sylvis primaevis propè vicum Aguassu, haud longè ab urbe Rio de Janeiro. Florebat Februario [1816–1821, *A. De Saint-Hilaire s/n*] (P! P00093861; isolectotypes: MPU 2-sheets MPU014277, MPU014278). (Fig. 3)
= Tovomitafoliosa C.Presl, Symb. Bot. (Presl) ii(7). 20. tab. 66. 1834 (1833). Type. lectotype (designated here), illustration in Presl (1834: Tab. 66). (Fig. 4)

#### Type.

lectotype (designated here), illustration in Sprengel (1821: Tab. I). (Fig. 1, 1–4b)

#### Notes about *Tovomitopsissaldanhae*

*Tovomitopsissaldanhae* was described by Engler (1888) based on specimens from the Serra dos Órgãos (Rio de Janeiro state) as “*Habitat in Brasiliae provincia Rio de Janeiro, in Serra dos Orgâos ad Theresopolim: J. de Saldanha n. 6777, 6780, 6781, Glaziou n. 13576 in herb. Eichler*”. His description and the illustration point to the use of both staminate and pistillate specimens. In the same contribution, Engler also described *Clusiaangustifolia* Engl. based on *Saldanha 7335*, which was collected in the same locality of *T.saldanhae*. The specimen (pistillate) clearly matches with specimens of *T.saldanhae*, especially the oblanceolate leaves with dark dots below, four petals and two series of resiniferous staminodes with subapical antherodes. As the specimen of *Clusiaangustifolia* deposited in B was destroyed during World War II in 1943, we select a duplicate at R as the lectotype.

**Figure 5.**
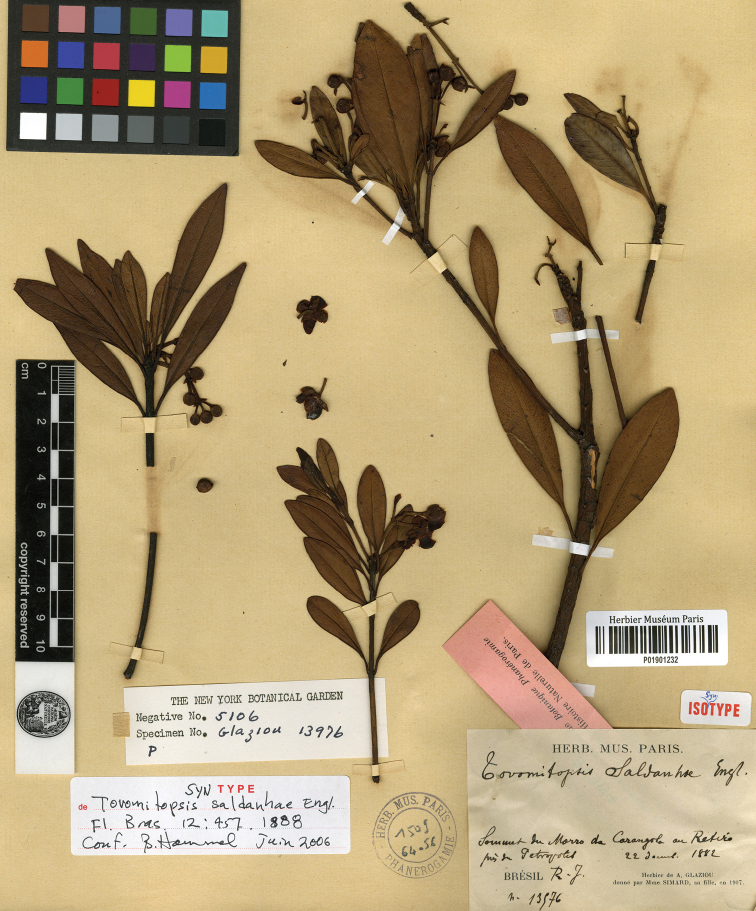
Lectotype of *Tovomitopsissaldanhae* Engl. (P01901232) housed at P.

*Tovomitopsissaldanhae* was later transferred to *Chrysochlamys* as *C.saldanhae* (Engl.) Oliveira-Filho, but Oliveira-Filho (2006) did not mention or indicate a type. *Chrysochlamyssaldanhae* is now a synonym of *T.saldanhae* (Marinho 2021). Among the *T.saldanhae* syntypes, we chose *A. Glaziou 13576* (P01901232, Fig. 5), which is the best preserved specimen housed at P, as the lectotype.

### Tovomitopsis saldanhae

Taxon classificationPlantaeMalpighialesClusiaceae

Engl., Fl. Bras. (Martius) 12(1): 457. 1888.

F51DBBCC-C78C-5E36-A4CC-35A2961E323B

**Figure 6.**
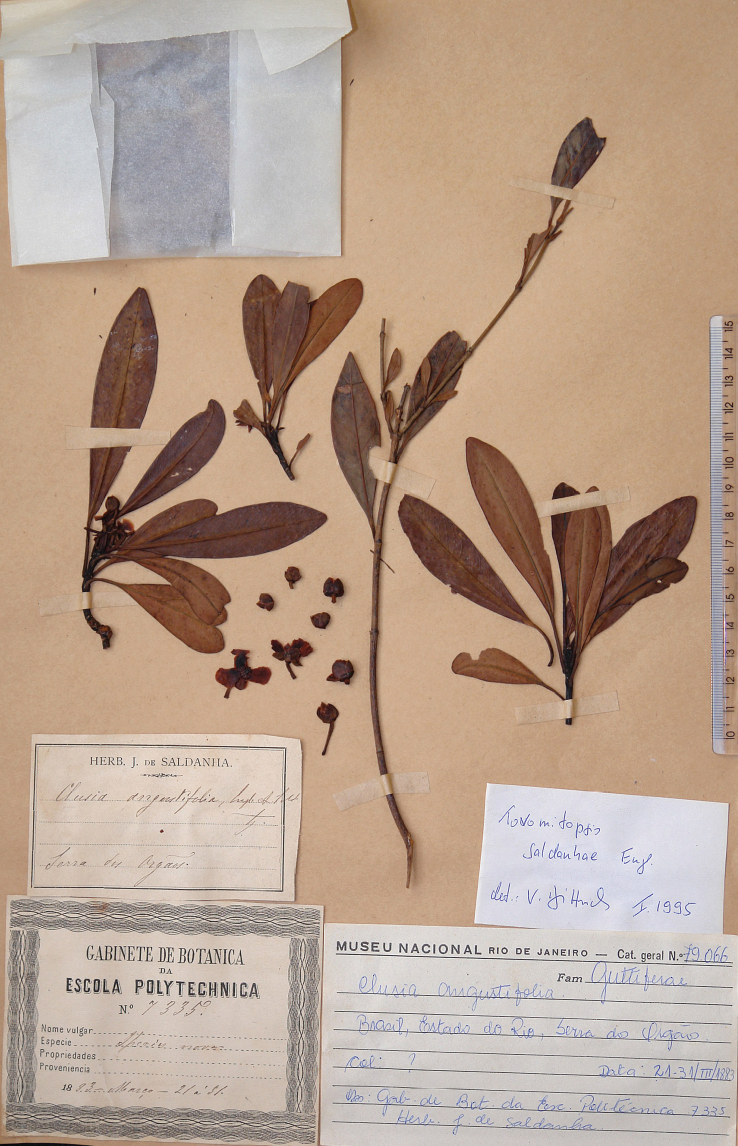
Lectotype of *Clusiaangustifolia* Engl. housed at R. The long branch in the center belongs to a different species not yet identified. Photographed by V. Bittrich.

≡ Chrysochlamyssaldanhae (Engl.) Oliveira-Filho, Cat. Árvores Nativas Minas Gerais: 93. 2006.
= Clusiaangustifolia Engl., Fl. Bras. (Martius) 12(1): 420. 1888, syn. nov. Type. lectotype (designated here), [Brazil: Rio de Janeiro] *habitat in Brasiliae provincia Rio de Janeiro, in Serra dos Orgâos* [21–31 March 1883], *J. Saldanha da Gama 7335* (R! excl. branch with long internodes in the center and leaves without dark dots below). (Fig. 6)

#### Type.

lectotype (designated here), [Brazil: Rio de Janeiro, Petrópolis], *Sommet du Morro da Carangola an Retiro, près de Petropolis*, [22 December 1882], *A. Glaziou 13576* (P! P01901232; isolectotypes: BR BR0000008675873, F F0360328F, P! P01901230, P! P01901231, R R000007580).

#### Note.

Saldanha’s collection numbers listed in a close numerical sequence may well belong to the same gathering (R. Forzza pers. com.). However, it is not possible for us to authenticate the preceding.

Regarding the other species of *Tovomitopsis* described by Engler (1888), Vesque (1893) transferred *Tovomitopsisspruceana* to *Clusia* (as *C.trochiformis* Vesque) and inadvertently proposed the lectotype by citing *Spruce 4569* at the Boissier herbarium (now housed at G) as the type, but unfortunately this specimen was not found.

### Emended description of *Tovomitopsis*

#### Tovomitopsis

Taxon classificationPlantaeMalpighialesClusiaceae

Planch. & Triana, Ann. Sci. Nat., Bot., sér. 4, 14: 261. 1860.

A7007FC2-236C-5B04-AE1C-875226AA8289

≡ Bertolonia Spreng., Neue Entdeck. Pflanzenk. 2: 110. 1820 (‘1821’), non Raddi (1820)

##### Type.

*Tovomitopsispaniculata* (Spreng.) Planch & Triana. (*Bertoloniapaniculata* Spreng.)

##### Description.

**Figure 7.**
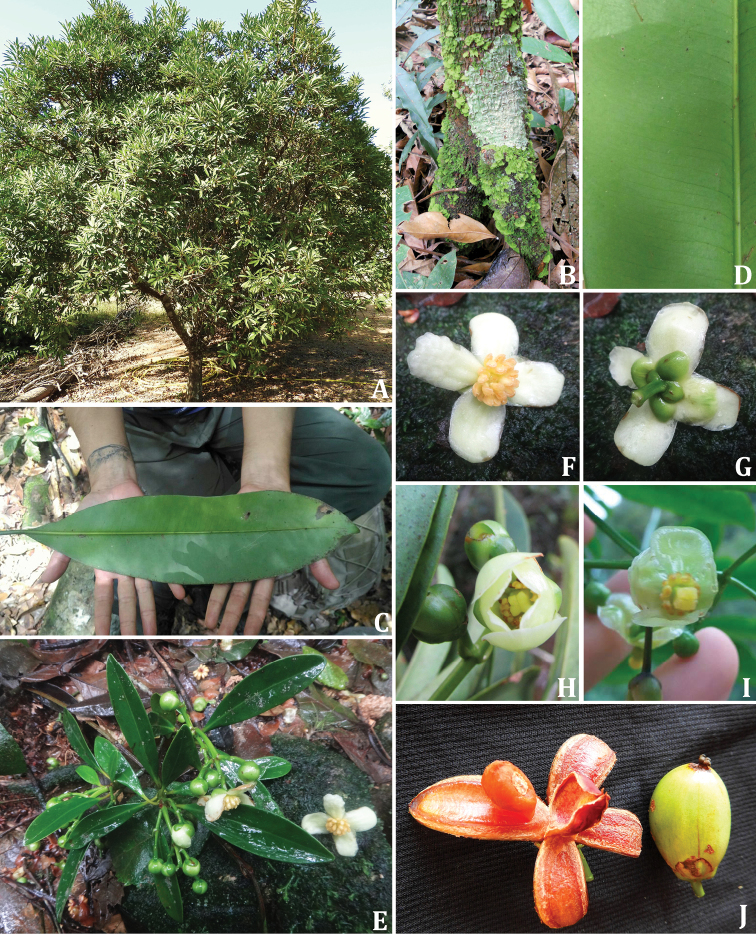
General morphology of *Tovomitopsis*. **A** habit **B** prop roots; **C** leaf undersurface **D** detail of leaf showing the veins **E** branch with leaves and staminate flowers **F, G** staminate flower **H** pistillate flower in the beginning of anthesis **I** pistillate flower in anthesis **J** open (left) and closed fruit (right). **A–D, I, J***T.paniculata***E–H***T.saldanhae*. Photos **A** and **J** Marcelo Mig **B** Lucas Marinho **C, D** Ana Cláudia Alencar **E–G** Rodrigo Penati **H** Luciano Pedrosa **I** Rodrigo Castro.

Small dioecious trees or shrubs with prop roots; axillary shoots with internodes regularly spaced from each other, grouped at the branch apex; exudates yellowish viscous on the branches and leaves. Leaves simple, opposite, decussate, petiolate; leaf blades chartaceous or coriaceous, light green *in vivo*, greenish to grayish *in sicco*, margin entire; venation simple brochidodromous, midvein prominent abaxially, flat adaxially; secondary veins slightly arcuate or straight, prominent abaxially, flat adaxially, forming angles between 40°–65° with the midvein; major secondary spacing generally regular; intersecondary veins parallel to major secondary veins, one per intercostals area; intramarginal secondary veins sometimes present. Inflorescences terminal, cymose, widely lax or congested, a single dichasium or a closed thyrse (the staminate more floriferous than the pistillate); bracteoles 2, triangular. Buds green, spheroid, apex rounded; sepals 2 pairs, green, decussate, base truncate, margin entire, apex rounded, outer pair smaller than inner pair and not enclosing the bud; petals 4, whitish, base truncate, margin entire, apex rounded. Staminate flowers with ca. 25 resiniferous stamens, filaments dorsiventrally compressed, yellow, sometimes the outer ones smaller than the inner ones, anthers lateral, yellow, thecae with longitudinal dehiscence, resiniferous glands present at the dorsal side of the anthers, pistillode inconspicuous. Pollen with general format in polar view subtriangular; isopolar, tricolporate, and with long ectocolpi; reticulate, and the semitectum not solid, but composed of twisted bacula. Pistillate flowers with staminodes similar to stamens; ovary 4-locular, 1 ovule per locule, styles 4, very short, distinct and persistent; stigmas 4, capitate, free from each other, persistent. Capsule pendant or straight on the branch, with 4 valves, epicarp smooth, green, mesocarp light red to purplish red. Seeds 1 per locule, each enclosed by a vascularized orange aril. Fig. 7.

### *Tovomitopsis* and *Chrysochlamys* relationship

The phylogenetic relationship between *Tovomitopsis* and *Chrysochlamys* remained unknown until DNA sequence data became available for most genera of Clusieae. Taxonomic errors of attributing new Central American species to *Tovomitopsis* instead of *Chrysochlamys* (Maguire 1977) were likely due to characterizing the latter as having cauliflorous inflorescences, a condition only observed in *Dystovomita* (Engl.) D’Arcy among Clusieae. Moreover, Maguire (1977) almost certainly did not look at the type species of *Chrysochlamys*, i. e. *C.multiflora* Poepp., and it is doubtful that he analyzed species of *Tovomitopsis* from southeastern Brazil, including the type *T.paniculata*. A few years later, D’Arcy (1980) synonymized *Chrysochlamys* under *Tovomitopsis*, even though the latter is more recent (Hammel 1999).

Recent phylogenetic analyses of Clusieae demonstrated that *Chrysochlamys* is not sister to *Tovomitopsis*, but to *Clusia* (Gustafsson et al. 2007; Ruhfel et al. 2011; Marinho et al. 2019), and that floral resin evolved more than once in Clusieae and, possibly, even in *Clusia* (Gustafsson et al. 2007; Ruhfel et al. 2011). However, few species of *Chrysochlamys* were sampled in these studies, and phylogenetic relationships in this genus remain uncertain. Moreover, in two species of *Chrysochlamys* with resiniferous androecia (*C.tenuifolia* Cuatrec. and *C.chrisharonii* Vásquez & R. Rojas), male plants produce resin in a “capitulum” in the center of the flowers, where stamens are basally inserted (Hammel 1999; Vásquez Martínez and Rojas González 2009), while this structure is absent in flowers of *Tovomitopsis*. Further phylogenetic studies are needed to investigate if these *Chrysochlamys* species with the uncommon androecial morphology really belong to the genus if would be better placed elsewhere.

## Final remarks

We present a brief contribution to the nomenclature of *Tovomitopsis*, a small endemic genus of Clusiaceae from the Brazilian Atlantic Forest. Except for the gross similarity with *Chrysochlamys*, the recognition of the genus is easy if associated with geographic distribution, as *Chrysochlamys* only occurs in Mexico, Central America and northern South America. Even so, further information on the geographic distribution and morphological limits between *T.paniculata* and *T.saldanhae* is needed. These two species are usually distinguished based on leaf size, shape and texture, but these features vary considerably both along an altitudinal gradient and from the coast to more inland sites. The presence and quantity of tiny blackish resinous glands on the abaxial surface of the leaves also should be considered and further investigated to distinguish species. An integrated taxonomic approach involving population genetics, geometric morphometrics of leaf outlines and classical taxonomy could shed light on species delimitations in *Tovomitopsis*.

## Supplementary Material

XML Treatment for Tovomitopsis paniculata

XML Treatment for Tovomitopsis saldanhae

XML Treatment for Tovomitopsis
